# Correlation Analysis Between Attentional Bias and Somatic Symptoms in Depressive Disorders

**DOI:** 10.3389/fpsyt.2019.00903

**Published:** 2019-12-13

**Authors:** Yun Wang, Yajun He, Gaohua Wang, Jiangbo Li, Haibing Zhu

**Affiliations:** ^1^Department of Psychiatry, Guangzhou Panyu Central Hospital, Guangzhou, China; ^2^Mental Health Centre, People’s Hospital of Wuhan University, Wuhan, China; ^3^Department of Pathology, Shenzhen Baoan District People’s Hospital, Shenzhen, China; ^4^Department of Clinical Psychology, Second People’s Hospital of Wuhu, Wuhu, China

**Keywords:** depressive disorder, attentional bias, *TORAWARE* state, depression, somatic discomfort

## Abstract

**Objective:**

To investigate the relationship between attentional bias and the severity of depression as assessed by the *TORAWARE* state and physical symptoms.

**Methods:**

We enrolled 55 patients with depression and 60 healthy people. The Hamilton Depression Scale (HAMD-24), Somatic Self-Rating Scale (SSS), and the Chinese version of the Self-Rating Scale for the *TORAWARE* State of Neurosis (SSTN) were selected to assess the severity of psychological symptoms. Dot-probe tasks were used to detect attentional bias. We then analyzed the correlation of attentional bias with the total scores on the symptom scales.

**Results:**

The negative attentional bias and negative disengaging index scores were both greater than 0 (t = 3.15 and 2.78, respectively; all P < 0.01). The negative attention bias score was positively correlated with the SSTN and negative disengaging index scores (r = 0.29 and 0.53, respectively; all P < 0.05). SSTN score was positively correlated with the total HAMD and SSS scores (r = 0.34 and 0.38, respectively; all P < 0.05).

**Conclusion:**

There is no direct correlation between negative attentional bias and depression. It may be through the intermediate mechanism of *TORAWARE* state to influence symptoms.

## Introduction

Approximately one third of patients with depression experience chronic or recurrent symptoms (1). In a previous study by Geden, 69% of patients with depression reported experiencing somatic symptoms only, while 11% denied experiencing depressive symptoms even when directly questioned regarding such symptoms (2). Additional studies have indicated that up to 50% of patients with depressive tendencies may be misdiagnosed due to the manifestation of physical/somatic symptoms rather than typical depressive symptoms (3, 4). Typical symptoms of depression include low mood, pessimism, feelings of meaninglessness, decreased energy, fatigue, low self-evaluation, guilt, and physical or biological symptoms such as loss of appetite and sleep disturbance. Previous meta-analyses have suggested that painful physical symptoms can be an important part of depression (5). Indeed, physical symptoms such as fatigue, weakness, chronic pain, and gastrointestinal disturbances (6, 7) often mask depressive symptoms, complicating the diagnosis and treatment of depression. Recent studies have demonstrated that rates of improvement in depressive symptoms are lower in patients with depression who exhibit somatic symptoms or comorbid physical illness than in those without comorbidities (8, 9). In addition, patients with poor physical health experience higher rates of relapse following recovery from the initial depressive episode. Evidence suggests that the degree of relief from somatic symptoms such as chronic pain is directly associated with higher rates of remission among patients with depressive symptoms (10). Such findings highlight the importance of treating somatic and physical symptoms associated with depression.

According to the cognitive model of depression, patients with depression exhibit a negative attentional bias that is manifested by their prioritization of negative cognitive stimuli. This attention bias of information processing plays a crucial role in the pathogenesis, maintenance, and development of depression (11, 12). One meta-analysis has suggested that patients with depression exhibit an attentional bias toward negative information (13). Negative attentional bias has been proven to predict the occurrence and recurrence of depressive symptoms (14, 15). Furthermore, patients with depression exhibit decreases in attentional bias toward positive information (16). Researchers have hypothesized that deficits in the processing of positive emotional information lead to decreases in the sensitivity to incentive stimuli, in turn leading to impairments in attentional orientation to such stimuli (17). In contrast, mentally healthy individuals exhibit attentional bias toward positive information, along with the tendency to avoid negative information (16). Prolonged absence of the attentional bias toward positive information leads to cognitive distortions (e.g., the attribution of negative events to oneself), exacerbating and maintaining depressive symptoms (18). However, the relationships among negative attentional bias, depressive symptoms, and physical discomfort in patients with depression remain unclear.

Dr. Morita Masaki, professor of Japanese psychiatry, first described the psychopathological characteristics of *TORAWARE*, a Japanese term referring to a state in which one’s attention and ideas are bound to a symptom or concept (i.e., neurotic preoccupation). *TORAWARE* is regarded as both a spiritual interaction and ideologically contradictory state (19, 20). Although patients may first experience conscious hyperprosexia, this state leads to decreases in the awareness of one’s surroundings, which in turn inhibits the ability to shift one’s attention, resulting in *TORAWARE* (21). Based on this theory, Morita therapy is a psychological therapy method invented by Japan, which is widely used in Asian countries and has significant clinical effects in treating neurosis, somatic symptoms of depression, generalized anxiety disorder, and other diseases (22).There are some studies on the application of Morita therapy, a research recruited 68 major depressive disorder participants (34 control and 34 intervention) provided 4-month follow-up data. We randomized participants on a 1:1 basis stratified by symptom severity to receive treat as usual (TAU) (control) or 8–12 sessions of Morita therapy plus TAU (intervention). Results compared with the control group, the symptom scale score of the control group was significantly improved. Morita therapy shows promise in treating depression and may provide patients with a distinct alternative to current treatments (23). He did a similar study before in 2016 (24). Morita therapists help patients to move away from symptom preoccupation and resistance, which are thought to interfere with the natural recovery process and lead to further preoccupation with and worsening of symptoms (25). Previous studies have demonstrated that pharmacological treatment combined with Morita therapy is more effective than treatment with medication alone, and that patients undergoing combined treatment exhibit more significant improvements in depressive symptoms and quality of life, along with fewer adverse effects (26, 27). While Morita therapy aims to break the cycle of attentional fixation on one’s symptoms, helping to accelerate improvements in both depressive and physical symptoms, some evidence suggests that depressive symptoms may also be present in the *TORAWARE* state (28).

In the nearly four years from 2015 to 2019 alone, a total of 180 papers on the application of Morita therapy have been retrieved from Chinese core journals. There must be a lot of studies in Japan, but there are few articles on Morita therapy in international scientific literature. After analyzing the reasons, we believe that: 1) apart from cultural factors, such as the difficult translation of its theory into English, it is not really easily understood by the international medical community; 2) the theory of Morita therapy is mainly derived from the summary of individual cases, and there are few large samples and experimental studies, so its theory does not exclude subjectivity and one-sidedness, and may lack of representativeness. So it didn’t get a lot of international attention. In order to avoid these disadvantages in the present study, we adopted the research method of medical psychology to discuss the attention characteristics of patients with depression and the correlation with somatic symptoms, and explore the occurrence and development mechanism of somatic symptoms of depression, and verify the scientific nature of Morita therapy theory. It is hoped that the international academic field has a new understanding of Morita therapy theory. According to the above theory, we hypothesized (a) that patients with depression would exhibit greater attentional bias toward negative emotional stimuli, as well as avoidance of positive stimuli; (b) that attentional bias toward negative emotional stimuli is positively correlated with the severity of depressive/somatic symptoms in patients with depression; (c) and that increases in negative attentional bias are associated with increases in the severity of the *TORAWARE* state and depressive/somatic symptoms.

## Patients and Methods

### Study Participants

The present cross-sectional study included 55 patients with depression and 60 healthy controls. Depression patients were recruited from the Clinical Psychology Department at Wuhu City Second People’s Hospital (Wuhu, China). Control participants with no current or prior psychiatric disorders were recruited from the Physical Examination Center. All prospective participants with depression were diagnosed by their treating psychiatrists using the Mini-International Neuropsychiatric Interview (MINI) in accordance with criteria outlined in the Diagnostic and Statistical Manual of Mental Disorders (DSM-IV) (29). The MINI is used to evaluate the mental state of depressed patients, including the degrees of cognitive impairment, depression and its duration, self-worth, and deterioration of physical and physiological states.

Inclusion criteria were as follows: (i) ≥15 years of age; (ii) all depression met the diagnostic criteria of DSM-IV for depressive disorder, Hamilton Depression Scale (HAMD-24) score >20; (iii) no history of organic brain disease, serious physical disease, or mental illnesses other than depression; (iv) no drug or alcohol use in the previous 2 weeks; (v) normal or corrected-to-normal vision. Exclusion criteria were as follows: (i) color blindness or presence of concurrent eye disease; (ii) history of alcohol/drug dependence; (iii) comorbid neuropsychiatric disorders; (iv) severe depression inhibiting completion of the study. All study participants provided written informed consent. The study was approved by the ethics committee at Wuhu Second People’s Hospital, Wannan Medical College (Wuhu, China).

### Self-Report Measures

The Hamilton Depression Scale (HAMD) was developed by Hamilton in 1960 and was the most commonly used scale in clinical evaluation of depression. This scale was conducted by two trained evaluators to conduct HAMD joint examination on patients. Generally, the method of conversation and observation was adopted. After the examination, the two evaluators scored independently. The severity and therapeutic effect of the disease can be evaluated by comparing the scores before and after treatment. The Somatic Self-Rating Scale (SSS) is a highly valid and reliable self-report questionnaire that is primarily used to evaluate emotional responses and somatic symptoms in patients treated at general hospitals (30). The SSS consists of 20 items divided into four domains, although we primarily utilized the somatization (S) factor to assess the severity of somatic symptoms in the present study. The Japanese version of the “Self-Rating Scale for the *TORAWARE* State of Neurosis” (SSTN) (31) was translated into Chinese in 2016 (32), exhibiting good internal consistency (α = .81). The SSTN is used to evaluate the severity of the *TORAWARE* state and consists of 20 items across the following six dimensions: mental interaction, mental conflict, attentional fixation, low social and body functioning, poor symptom tolerance, and perfectionism. Participants were asked to provide responses regarding symptoms experienced during the previous week using a four-point scale. Higher total scores were considered indicative of more severe symptoms.

### Dot-Probe Task

#### Materials

Forty positive, negative, and neutral pictures each were selected from the Chinese Affective Picture System (CAPS) (33). Positive and negative images were matched according to the degree of pleasure and polarization: The average pleasure rating of positive pictures was 7.68 ± 0.45, with an average degree of arousal of 5.72 ± 0.56. The average pleasure rating of negative pictures was 3.20 ± 0.35, with an average degree of arousal of 4.32 ± 0.66. Average pleasure and arousal ratings for neutral pictures were 5.34 ± 0.21 and 4.53 ± 0.34, respectively. Each picture was converted to a Bitmap (BMP) image with a resolution of 480 × 250 pixels using image editing software. Images were matched into negative–neutral, positive–neutral, neutral–neutral three types of combinations while taking the brightness and tonal proximity of the picture into consideration. The visual probe task was programmed using the E-prime software package, which was also used to present the experimental stimuli. Response times (RTs) and error rates were automatically recorded by the software, and the task was run on a computer with a 14-inch color monitor (resolution: 1366 × 768 pixels).

#### Procedures

All participants were tested in individual sessions lasting approximately 30 min each. Participants first completed the questionnaires, following which the experiment was conducted.

The dot-probe task consisted of 108 test trials (20 neutral–neutral, 40 negative–neutral trials, and 40 positive–neutral trials) and eight practice trials. Neutral and emotional picture pairs and probes appeared equally often to the left or right. Participants were seated at a viewing distance of approximately 60 cm from the computer screen, which displayed the experimental instructions. At the beginning of each trial, a fixation cross (“+”; 10 mm × 10 mm) was presented in the middle of the screen for 500 ms, following which two pictures (each 80 mm × 100 mm) were presented symmetrically on either side of the middle line of the screen for 500 ms. The distance between the inner edges of the pictures was 44 mm. Directly after the offset of the two pictures, a small probe point (“*”; 10 mm × 10 mm) appeared in the center of the location of either the left or right picture. Participants were asked to respond as quickly as possible by identifying the location of the probe and pressing one of two keys: the “A” key for left and the “L” key for right. The dot remained in view until participants provided a response or did not respond within 2,000 ms. A blank gray screen was presented for 1,000 ms prior to the onset of the next trial. Pictures were presented in random order.

### Analysis Indicators

Total HAMD-24, SSS-S, and SSTN scores, as well as attentional bias scores, were compared between the two groups. Bias scores were calculated by subtracting the mean RT for congruent trials from the mean RT for incongruent trials: bias score = RT_inconsistent_ – RT_consistent_. RT_inconsistent_ represents the average response time when the detection point was not in the same location as the emotional picture, while RT_consistent_ represents the average response time when the detection point was in the same location as the emotional picture. Positive scores are considered indicative of attentional bias towards emotional stimuli. We also calculated the orienting index (OI) and disengaging index (DI) (34) for each group, as follows: OI = RT_neutral_ _pair_ – RT_consistent_; DI = RT_inconsistent_ – RT_neutral_ _pair_. Positive OI values indicate that detection of emotional pictures occurred more quickly, while positive DI values indicate difficulty in disengaging attention from emotional pictures. Negative bias scores, OI values, and DI values were considered indicative of attentional avoidance of emotional pictures.

### Data Analysis

All statistical analyses were performed using SPSS version 20.0. After visual inspection of the data, RTs <200 ms and >1000 ms were considered outliers indicative of anticipatory and delayed responding, respectively. Trials with errors were discarded from the analyses, as we observed no significant differences in the number of erroneous responses between the two groups (F = 1.85, p > .1). Five participants with depression were excluded due to unusually slow RTs or the absence of 40% of RT data due to errors and outliers. Average individual RTs were computed for each emotion in each of the five conditions. Chi-square tests and *t*-tests were used to investigate between-group differences in demographic characteristics and symptom scores. One-sample *t*-tests were used to compare bias scores, OI values, and DI values to 0 (indicating a lack of attentional bias). Pearson correlation analyses were used to explore the relationships between attentional bias indices and symptoms.

## Results

### Participant Characteristics

As shown in **Table 1**, participants in the depression group reported significantly greater symptoms of depression and somatic discomfort than those in the control group (*t* [62.97] = 29.69, *t* [69.17] = 10.14; all P < 0.001). In addition, symptoms of the *TORAWARE* state were significantly greater in the depression group than in the control group (*t* [89.38] = 17.26; P < 0.001). No significant differences in age (*t* [113] = –1.3; P = 0.20), education (*t* [109.11] = –1.3; P = 0.07), or gender ratio (χ^2^ [1, *n* = 50] = 1.24; P = 0.27) were observed between the groups.

**Table 1:** Demographic information and participant test scores.

	Depression (*n* = 55)	Control (*n* = 60)
	Mean (SD)	Mean (SD)
Age	25.38 (8.7)	27.43 (8.19)
Sex (n; male/female)	14/41	21/39
Education (years)	13.56 (3.05)	14.58 (2.96)
HAMD-24	33.98 (7.40)	3.15 (2.23)
SSS-S	19.40 (5.55)	11.28 (2.19)
SSTN	57.73 (9.46)	32.03 (5.94)

SD, standard deviation; HAMD-24, 24-item Hamilton Depression Scale; SSS-S, Somatic Self-rating Scale–Somatic domain; SSTN, Self-rating Scale for the TORAWARE State of Neurosis.

### Attentional Indices for Positive and Negative Pictures

We compared the bias scores, OI values, and DI values for positive and negative stimuli to a no-bias criterion (zero) in each group. Statistically significant difference in negative attention bias score and negative DI in the control group (t = −4.18 and −2.19, respectively; P = 0.000 and 0.033, respectively), indicating that the control group exhibited avoidance of negative stimuli; while these two indicators were both significant >0 in the depression group (*t* = 3.15 and 2.78, respectively; P *=* 0.003 and 0.008, respectively), indicating that patients with depression exhibited attentional bias towards negative images as well as difficulty disengaging their attention from such stimuli. The positive bias score of the depression group was statistically significant (t = −2.29, P = 0.026), indicating that the participants in the depression group avoided positive stimuli (**Table 2**).

**Table 2:** Bias score, orienting index, and disengagement index (s) for positive and negative stimuli [(x ± s)/ms)].

Group	Bias score		Orienting index		Disengagement index	
	Negative	Positive	Negative	Positive	Negative	Positive
Depression	11.87 ± 27.93^**^	−8.43 ± 27.26^**^	−1.24 ± 31.27	−6.68 ± 31.15	13.1 ± 34.99^**^	−1.75 ± 33.19
Control	−13.75 ± 25.46**	−2.23 ± 31.03	−5.67 ± 24.48	−0.87 ± 30.21	−8.08 ± 28.64^*^	−1.36 ± 30.69

Compared with zero; * indicates a significant difference (p < 0.05); ** indicates a significant difference (p < 0.01).

### Relationships Between Attentional Indices and Symptoms in the Depression Group

We conducted a series of bivariate correlation analyses to explore whether attentional bias scores were associated with the severity of depressive or somatic symptoms. We further investigated the interaction among attentional bias, the *TORAWARE* state, and symptoms among patients with depression. **Table 3** shows the zero-order correlation coefficients. Our analyses revealed that the positive bias scores have no correlation with other indicators, negative bias scores were positively associated with negative DI values (r = 0.53, P < 0.001) and SSTN scores (r = 0.29, P = 0.035). SSTN scores were positively associated with HAMD-24 and SSS-S scores (r = 0.34 and 0.38, respectively; P = 0.011 and 0.005, respectively). No other significant associations were observed (**Table 3**).

**Table 3:** Bivariate correlations between attentional bias indices and symptoms in the depression group.

	Positive bias score	Negative bias score	Negative disengagement index	SSTN
Negative bias score	−0.242			
Disengagement index	−0.081	.53**		
SSTN	−0.059	.29*	.20	
HAMD-24	−0.043	.06	−.01	.34*
SSS-S	−0.118	−.01	.09	.38**

HAMD-24, 24-item Hamilton Depression Scale; SSS-S, Somatic Self-rating Scale–Somatic domain; SSTN: Self-rating Scale for the TORAWARE State of Neurosis. N = 55; * indicates a significant difference (p < 0.05); ** indicates a significant difference (p < 0.01).

## Discussion

Although scientists are trying to study “medical unexplained symptoms” (MUS) disease etiology, mechanisms, but so far MUS is still a difficult problem in the international medical. Most depression patients with MUS, like often accompanied by physically weakening symptoms, such as loss of appetite, fatigue, and weight loss. This results in a reluctance to partake in social activities, which further affects the patient’s relationships. Many patients’ somatoform discomforts disappeared after the depression cured, but why depression are prone to body symptom? We found that Morita therapy can achieve good results in the treatment of somatic symptoms of depression, and the psychological mechanism is worth studying. Clinical depression is generally found to have negative thinking tendency (looking at negative aspects of things and ignoring positive aspects). Is this thinking tendency related to debilitating symptoms? In the present study, we investigated the associations among attentional bias, the *TORAWARE* state, and somatic/depressive symptoms in patients with clinical depression. Our findings indicated that patients with depression exhibit obvious attentional bias towards negative image information, which is mainly manifested as difficulty in disengaging attention, consistent with the results of previous studies (35, 36). In addition, control participants exhibited attentional avoidance with regard to negative stimuli, consistent with previous findings as well as our first hypothesis. Previous research has suggested that such avoidance exerts a protective effect in healthy individuals confronted with negative stimuli (37); however, excessive processing occurs in patients with depression, making it difficult to refocus attention and increasing vulnerability to negative information (25).

Saskia et al. (38) explored the relationship between attentional bias and the severity/specificity of symptoms in patients with depression using the Beck Depression Inventory-II (BDI-II), which evaluates the following three dimensions of depression: negative cognition, depressive symptoms, and somatic symptoms. The authors demonstrated that only negative cognition is a main predictor of negative attentional bias among patients with depression. Disner et al. (39) reported that negative attentional bias is related to the course of depressive symptoms, although they observed no significant relationship between such bias and baseline levels of depressive symptoms. Such results suggest that there is no direct correlation between negative attentional bias and depressive symptoms in patients with depression, in accordance with our findings. Although we observed no direct correlation between negative attentional bias and somatic discomfort or depressive symptoms, such bias was positively correlated with the severity of the *TORAWARE* state, and the *TORAWARE* state was positively correlated with the severity of symptoms. These findings suggest that the *TORAWARE* state of patients with depression may play a mediating role in the effect of attention bias of negative stimuli on depressive and somatic symptoms.

The current hypothesis regarding the pathology of the *TORAWARE* state proposes that patients with neuroses exhibit cognitive biases, which Morita referred to as “ideological contradictions”. Such biases are thought to lead to involuntarily or consciously excessive attention (attentional fixation) on symptoms such as physical discomfort or annoyance, thereby exacerbating these feelings. This in turn leads to involuntary fixation on or hypersensitivity to such symptoms, decreasing social functioning and maintaining the *TORAWARE* state (20, 28). The somatic discomfort experienced by patients with depression is often similar to neurosis in that patients may excessively focus on physical discomfort and depressive symptoms. In the present study, we observed that SSTN scores were significantly higher among patients with depression than in the control group, suggesting that the psychopathology of depression can also involve the *TORAWARE* state. We also observed a significant positive correlation between negative attentional bias and the severity of the *TORAWARE* state in the depression group. Furthermore, the severity of the *TORAWARE* state was significantly positively correlated with depression and somatosensory symptom scores, in accordance with our hypotheses. These results suggest that attentional bias toward negative information in patients with depression can reinforce depressive symptoms and somatic discomfort, and that this process is mediated by the *TORAWARE* state. Moreover, the negative DI value in the depression group was significantly larger than 0, consistent with the results of Zhu et al. (40). These results suggest that patients with depression exhibit difficulty disengaging from negative stimuli, which may underlie attentional fixation. Such attentional fixation may aggravate depressive/somatic symptoms, thereby leading to the development and maintenance of the depressive state.

Our findings support the notion that patients with depression exhibit negative attentional bias. Our results further suggest that the presence of depressive and somatic symptoms may lead to the development of attentional bias and the *TORAWARE* state. The results of our study are exciting. It is the first time to demonstrate the mechanism of the *TORAWARE* state in role of depression with somatic symptoms by experimental method, and it also helps us find a new target for the treatment of somatic symptoms of depression, which is beneficial to the research and treatment of somatic symptoms of depression. Further studies are required to determine whether resolution of the *TORAWARE* state can improve depressive/somatic symptoms in patients with depression. Our research subjects are limited to Chinese patients with depression. Due to cultural and ethnic factors, our conclusions may be limited or lack of representation in different ethnic and cultural backgrounds worldwide. However, one fourth of the world’s population in China has such research conclusion on depression, which may suggest that this study has important reference value for the worldwide exploration of somatic symptoms of depression or the pathogenesis of MUS.

## Ethics Statement

This study was carried out in accordance with the recommendations of Ethical guidelines for biomedical research involving people, Wuhu Second People's Hospital Ethics Committee. The protocol was approved by the Wuhu Second People's Hospital Ethics Committee. All subjects gave written informed consent in accordance with the Declaration of Helsinki.

## Author Contributions

The completion of the experiment and the writing of the article were mainly by YW. YH helped with data processing and analysis, and proofread the manuscript. GW helped strictly check the quality of the article and his project fund supported the successful conduct and completion of the research. LJ helped with corrections and revisions of this paper and also have given me a lot of advice on the shortcomings of the article. HZ provided partial fund support and academic guidance.

## Funding

This project received funding from the Panyu District Science and Technology Plan Project (Grant No.: 2017-Z04-02) and Mental Health Okamoto Memorial Consortium Research Support Project, Pingcheng 29 years graduate 8. NSFC: Study on the role and mechanism of NLRP3 inflammatory cortisone/il-18/NF-B neuroinflammatory pathway in mediating the occurrence and outcome of depression, and 81871072 to GW.

## Conflict of Interest

The authors declare that the research was conducted in the absence of any commercial or financial relationships that could be construed as a potential conflict of interest.
