# Corrigendum: Correlation Analysis Between Attentional Bias and Somatic Symptoms in Depressive Disorders

**DOI:** 10.3389/fpsyt.2020.00293

**Published:** 2020-04-24

**Authors:** Yun Wang, Yajun He, Gaohua Wang, Jiangbo Li, Haibing Zhu

**Affiliations:** ^1^ Department of Psychiatry, Guangzhou Panyu Central Hospital, Guangzhou, China; ^2^ Mental Health Centre, People’s Hospital of Wuhan University, Wuhan, China; ^3^ Department of Pathology, Shenzhen Baoan District People's Hospital, Shenzhen, China; ^4^ Department of Clinical Psychology, Second People’s Hospital of Wuhu, Wuhu, China

**Keywords:** depressive disorder, attentional bias, TORAWARE state, depression, somatic discomfort

In the original article, we neglected to include the following funding, “NSFC: Study on the role and mechanism of NLRP3 inflammatory cortisone/il-18/NF-B neuroinflammatory pathway in mediating the occurrence and outcome of depression, and 81871072 to GW.”

Yajun He was not included as an author in the published article. In addition, Gaohua Wang should be included as a co-correspondence author in the published article. The Author Contributions statement has been corrected and appears below.

## Author Contributions

“The completion of the experiment and the writing of the article were mainly by YW. YH helped with data processing and analysis, and proofread the manuscript. GW helped strictly check the quality of the article and his project fund supported the successful conduct and completion of the research. LJ helped with corrections and revisions of this paper and also have given me a lot of advice on the shortcomings of the article. HZ provided partial fund support and academic guidance.”

The authors apologize for these errors and state that this does not change the scientific conclusions of the article in any way. The original article has been updated.

